# Crystal structure of bis­(aceto­nitrile-κ*N*)(4,4′-di-*tert*-butyl-2,2′-bi­pyridine-κ^2^ *N*,*N*′)platinum(II) bis­(tetra­fluorido­borate) packing as head-to-head dimers

**DOI:** 10.1107/S2056989018005923

**Published:** 2018-04-24

**Authors:** Chris Joseph, Vladimir N. Nesterov, Bradley W. Smucker

**Affiliations:** a Austin College, 900 N Grand, Sherman, TX 75090-4400, USA; b University of North Texas, 1155 Union Circle, Denton, TX 76203-5070, USA

**Keywords:** supra­molecular, platinum(II), crystal structure

## Abstract

The crystal structure of a platinum(II) supra­molecular building block, [Pt(dbbpy)(NCCH_3_)_2_](BF_4_)_2_ (dbbpy = 4,4′-di-*tert*-butyl-2,2′-bi­pyridine, C_18_H_24_N_2_) is an example of a rare head-to-head dimer, even with the bulky *tert*-butyl groups of the bi­pyridine. This packing motif still enables significant π–π inter­actions between two pyridyl groups, and may result from the close proximity of the tetra­fluorido­borate ions to the platinum(II) complexes, resulting in intra­molecular H⋯F distances between 2.156 and 2.573 Å.

## Abstract 2

The bulky *tert*-butyl groups of the dbbpy ligand do not preclude the formation of head-to-head dimers in the crystal structure of bis­(aceto­nitrile)(4,4′-di-*tert*-butyl-2,2′-bi­pyridine)­platinum(II) tetra­fluorido­borate.

## Chemical context   

The title compound is soluble in a diverse range of solvents and possesses exchangeable aceto­nitrile ligands for facile incorporation of novel ligands to develop new and diverse behaviors of platinum(II) complexes. The solubility and apt geometry of the (dbbpy)platinum(II) complex make it a desirable building block for coordination-driven self-assembly of homo-metallic (Zhang, *et al.*, 2017 ▸) and hetero-metallic (Bera *et al.*, 2001 ▸) supra­molecular complexes. This platinum(II) di­imine can also be combined with di­thiol­ene ligands to study methyl­ation kinetics (Stace, *et al.*, 2016 ▸), generate charge-transfer materials (Smucker, *et al.*, 2003 ▸), or make model complexes for examining photophysical properties (Laza­rides, *et al.*, 2011 ▸; Yang *et al.*, 2014 ▸).
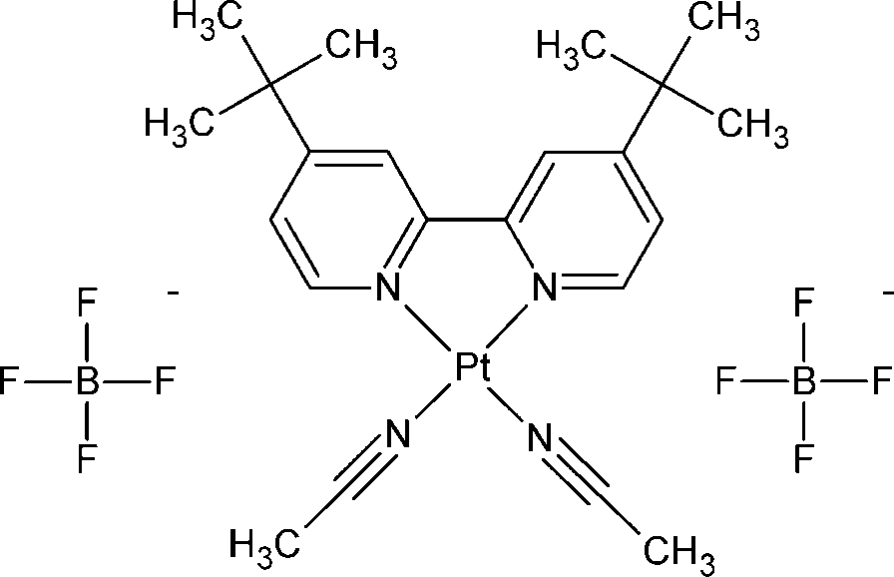


## Structural commentary   

The platinum–nitro­gen distances for the bipyridyl N1 and N2 of the +2 cation are 1.994 (4) and 1.995 (4) Å, respectively, with a bond angle of 80.5 (2)°. These are shorter than those affected by the stronger *trans*-influence of chloride in two structures of the neutral (dbbpy)PtCl_2_ mol­ecule: one with Pt—N distances of 2.013 (2) and 2.011 (2) Å and a 79.79 (6)° N—Pt—N angle (Day, 2009 ▸), and the other having Pt—N distances of 2.010 (12) and 2.019 (10) Å and a 78.7 (5)° N—Pt—N angle (Achar & Catalano, 1997 ▸). The Pt—N distances of the title compound are longer than those having the weaker *trans*-influence of water in the +2 cation of [(dbbpy)Pt(OH_2_)_2_](OTf)_2_ (Singh *et al.*, 2008 ▸), which exhibits Pt—N distances of 1.966 (5) and 1.974 (5) Å and the resulting wider bond angle of 81.1 (2)° for N—Pt—N. The *trans*-influence of the ligand is, indeed, on par with that of the related mono-cation [(dbbpy)Pt(NCCH_3_)(Ph)] [BAr’_4_], containing a Pt—N distance of 2.000 (4) Å, located *trans* to the aceto­nitrile, while the phenyl ligand causes an elongation to 2.092 (4) Å for the other Pt—N bond (McKeown, *et al.*, 2011 ▸).

## Supra­molecular features   

**Figure 1:**
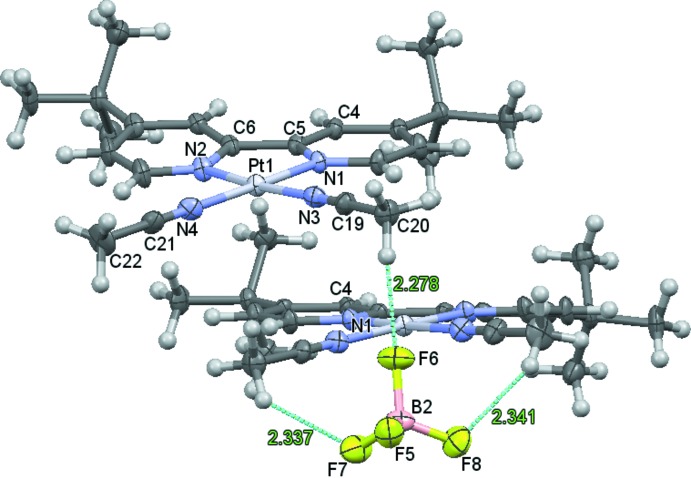
Displacement ellipsoid plot (50% probability of all non-H atoms), illustrating the head-to-head dimer with selected H⋯F inter­molecular distances (Å) between a BF_4_^−^ anion and aceto­nitrile mol­ecules on adjacent mol­ecules.

**Figure 2:**
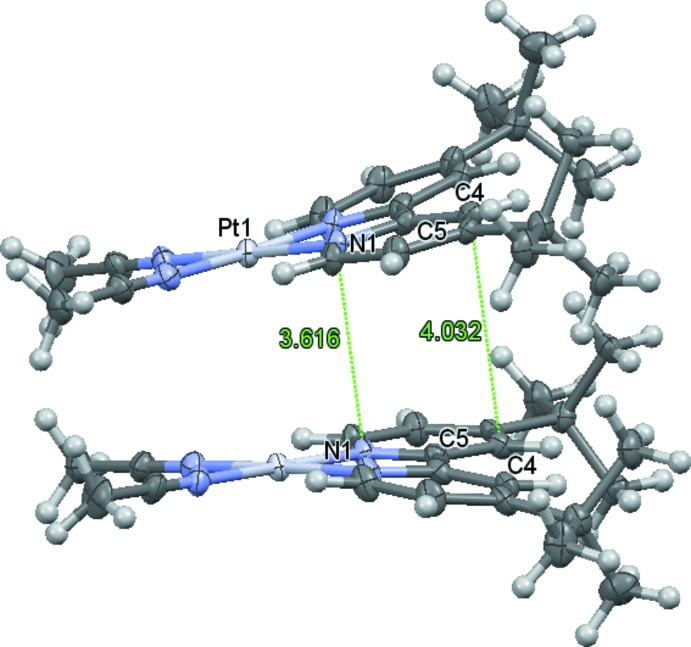
Displacement ellipsoid plot (50% probability of all non-H atoms), illustrating the slightly canted head-to-head dimer with selected intra­molecular distances shown.

Most platinum(II) compounds containing the bulky dbbpy ligand pack as head-to-tail dimers, such as the aforementioned (dbbpy)PtCl_2_ (Day, 2009 ▸; Achar & Catalano, 1997 ▸), [(dbbpy)Pt(OH_2_)_2_](OTf)_2_ (Singh *et al.*, 2008 ▸), [(dbbpy)Pt(NCCH_3_)(Ph)][BAr′_4_] (Ar′ = 3,5-bis(tri­fluoro­methyl)phenyl; McKeown *et al.*, 2011 ▸), and (dbbpy)Pt(dmid) (dmid = 1,3-di­thiole-2-one-4,5-di­thiol­ate; Smucker *et al.*, 2003 ▸). The cations in the title compound, however, pack as head-to-head dimers (Figs. 1 ▸ and 2 ▸). In these dimers, the mol­ecules are offset (translation by half a mol­ecule)and slightly canted [the planes composed of all non-H atoms except the *tert*-butly groups for the (dbbpy)Pt(NCCH_3_)_2_ cation and its corresponding dimer (−*x*, *y*, 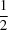 − *z*) are at an angle of 10.82°], both of which accommodate the bulky *tert*-butyl groups of the dbbpy ligands. The intra­molecular Pt—Pt distance is quite long at 4.5123 (3) Å, yet the pyridyl rings of the dbbpy are positioned for π–π inter­actions with distances between 3.616 (5) Å (N1⋯N1^i^) and 4.032 (7) Å (C4⋯C4^i^) [symmetry code: (i) −*x*, *y*, 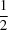 − *z*] occurring between the two rings (Fig. 2 ▸). This atypical head-to-head packing may be partly explained through the favorable non-polar inter­actions between the *tert*-butyl groups. Another viable explanation comes through the inter­molecular inter­actions between fluorine atoms of the BF_4_^−^ ions and the hydrogen atoms on the pyridine and aceto­nitrile ligands on multiple cations. Indeed, all eight fluorine atoms of the two unique BF_4_^−^ anions are in close proximity to hydrogen atoms on the cation with inter­molecular H⋯F distances between 2.16 and 2.57 Å (Fig. 1 ▸ and Table 1 ▸). Changing the anion in related bis­(aceto­nitrile)(di­imine) platinum(II) cations seems to have a significant influence, as observed in the structures of 2,2′-bi­pyridine in [(bpy)Pt(NCCH_3_)_2_](OTf)_2_ (Field *et al.*, 2003 ▸) or 1,10-phenanthroline in [(phen)Pt(NCCh_3_)_2_](ClO_4_)_2_ (Ha, 2010 ▸), which do not form dimers as the positions of the triflate or perchlorate anions minimize the close proximity of the two platinum-containing cations.

## Synthesis   

The synthesis of the title compound used a method which replaced the chloride from Pt(dbbpy)Cl_2_ (Tzeng *et al.*, 2001 ▸) with aceto­nitrile using excess AgBF_4_ by following the general syntheses of (dbbpy)Pt(SO_3_CF_3_)_2_ (Hill *et al.*, 1996 ▸) and [Pt(NCCH_3_)_4_](BF_4_)_2_ (de Renzi *et al.*, 1976 ▸).

**[Pt(dbbpy)(NCCH_3_)_2_](BF_4_)_2_** A solution containing 25 mL of aceto­nitrile, 200.7 mg (0.2500 mmol) of Pt(dbbpy)Cl_2_, and 164 mg (0.8425 mmol) of AgBF_4_ was refluxed under stirring until a yellow solution formed. The solution was isolated, *via* cannula, from the AgCl precipitate and condensed under reduced pressure until ∼5 mL of orange solution remained. This was combined with 25 ml of Et_2_O and the resulting precipitate was washed with 3 × 20 mL Et_2_O to give 206.9 mg (83.8% yield) of product. UV–vis λmax (∊ Lmol^−1^cm^−1^): 211 (4.6 × 10^4^), 249 (4.2 × 10^4^), 306 (2.0 × 10^4^), 319 (2.4 × 10^4^) and 346 (6.0 × 10^3^) nm.

Yellow crystals of the title compound were grown from liquid diffusion of hexa­nes into a dilute acetone solution.

## Refinement   

Crystal data, data collection and structure refinement details are summarized in Table 2 ▸. H atoms were attached to C atoms and ideally positioned (C—H = 0.95–0.98 Å) and refined as riding with *U*_iso_(H) = 1.2*U*_eq_(CH) or *U*_iso_(H) = 1.2*U*_eq_(CH_3_).

## Supplementary Material

Crystal structure: contains datablock(s) I. DOI: 10.1107/S2056989018005923/jj2198sup1.cif

Structure factors: contains datablock(s) I. DOI: 10.1107/S2056989018005923/jj2198Isup2.hkl

Supporting information file. DOI: 10.1107/S2056989018005923/jj2198Isup3.mol

CCDC reference: 1837532

Additional supporting information:  crystallographic information; 3D view; checkCIF report

## Figures and Tables

**Table 1:** Inter­molecular H⋯F distances (Å) between all eight fluorine atoms of the two BF_4_^−^ anions

F1⋯H1*A*	2.16	F5^iii^⋯H7*A* ^iv^	2.30
F1⋯H20*B*	2.43	F5^iii^⋯H4*A* ^iv^	2.35
F2⋯H9*A* ^ii^	2.43	F6^iii^⋯H20*C* ^i^	2.28
F3⋯H9*A* ^ii^	2.57	F7^iii^⋯H20*A*	2.34
F4⋯H2*A*	2.43 (4)	F8^iii^⋯H22*C*	2.34

Symmetry codes: (i) −*x*, *y*, 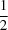 − *z*; (ii) *x*, 1 − *y*, 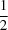 + *z*; (iii) 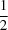 − *x*, 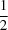 + *y*, *z*; (iv) *x*, 1 + *y*, *z*.

**Table 2:** Experimental details

Crystal data	
Chemical formula	[Pt(C_18_H_24_N_2_)(C_2_H_3_N)_2_](BF_4_)_2_
*M*_r_	719.21
Crystal system, space group	Orthorhombic, *P* *b* *c* *n*
Temperature (K)	100
*a*, *b*, *c* (Å)	16.3409 (10), 13.0447 (8), 25.1105 (16)
*V* (Å^3^)	5352.6 (6)
*Z*	8
Radiation type	Mo *K*α
μ (mm^−1^)	5.32
Crystal size (mm)	0.14 × 0.14 × 0.08

Data collection	
Diffractometer	Bruker APEXII CCD
Absorption correction	Multi-scan (*SADABS*; Bruker, 2001 ▸)
*T*_min_, *T*_max_	0.515, 0.682
No. of measured, independent and observed [*I* > 2σ(*I*)] reflections	61655, 5919, 4823
*R*_int_	0.048
(sin θ/λ)_max_ (Å^−1^)	0.641

Refinement	
*R*[*F* ^2^ > 2σ(*F* ^2^)], *wR*(*F* ^2^), *S*	0.033, 0.113, 1.01
No. of reflections	5919
No. of parameters	342
H-atom treatment	H-atom parameters constrained
Δρ_max_, Δρ_min_ (e Å^−3^)	1.39, −1.56

Computer programs: *APEX2* (Bruker, 2007 ▸), *SHELXS97* (Sheldrick, 2008 ▸), *SHELXL97* (Sheldrick, 2008 ▸), *SHELXTL* (Sheldrick, 2008 ▸) and *Mercury* (Macrae *et al.*, 2006 ▸).

## supplementary crystallographic information

### Crystal data

**Table:**

[Pt(C_18_H_24_N_2_)(C_2_H_3_N)_2_](BF_4_)_2_	*F*(000) = 2800
*M**_r_* = 719.21	*D*_x_ = 1.785 Mg m^−^^3^
Orthorhombic, *P**b**c**n*	Mo *K*α radiation, λ = 0.71073 Å
Hall symbol: -P 2n 2ab	Cell parameters from 9946 reflections
*a* = 16.3409 (10) Å	θ = 2.2–27.1°
*b* = 13.0447 (8) Å	µ = 5.32 mm^−^^1^
*c* = 25.1105 (16) Å	*T* = 100 K
*V* = 5352.6 (6) Å^3^	Plate, yellow
*Z* = 8	0.14 × 0.14 × 0.08 mm

### Data collection

**Table:**

Bruker APEXII CCD diffractometer	5919 independent reflections
Radiation source: fine-focus sealed tube	4823 reflections with *I* > 2σ(*I*)
Graphite monochromator	*R*_int_ = 0.048
ω scans	θ_max_ = 27.1°, θ_min_ = 1.6°
Absorption correction: multi-scan (SADABS; Bruker, 2001)	*h* = −20→20
*T*_min_ = 0.515, *T*_max_ = 0.682	*k* = −16→16
61655 measured reflections	*l* = −32→32

### Refinement

**Table:**

Refinement on *F*^2^	Primary atom site location: structure-invariant direct methods
Least-squares matrix: full	Secondary atom site location: difference Fourier map
*R*[*F*^2^ > 2σ(*F*^2^)] = 0.033	Hydrogen site location: inferred from neighbouring sites
*wR*(*F*^2^) = 0.113	H-atom parameters constrained
*S* = 1.01	*w* = 1/[σ^2^(*F*_o_^2^) + (0.080*P*)^2^ + 5.*P*] where *P* = (*F*_o_^2^ + 2*F*_c_^2^)/3
5919 reflections	(Δ/σ)_max_ = 0.001
342 parameters	Δρ_max_ = 1.39 e Å^−^^3^
0 restraints	Δρ_min_ = −1.55 e Å^−^^3^

### Special details

**Table:**

Geometry. All esds (except the esd in the dihedral angle between two l.s. planes) are estimated using the full covariance matrix. The cell esds are taken into account individually in the estimation of esds in distances, angles and torsion angles; correlations between esds in cell parameters are only used when they are defined by crystal symmetry. An approximate (isotropic) treatment of cell esds is used for estimating esds involving l.s. planes.
Refinement. Refinement of F^2^ against ALL reflections. The weighted R-factor wR and goodness of fit S are based on F^2^, conventional R-factors R are based on F, with F set to zero for negative F^2^. The threshold expression of F^2^ > 2sigma(F^2^) is used only for calculating R-factors(gt) etc. and is not relevant to the choice of reflections for refinement. R-factors based on F^2^ are statistically about twice as large as those based on F, and R- factors based on ALL data will be even larger.

### Fractional atomic coordinates and isotropic or equivalent isotropic displacement parameters (Å^2^)

**Table:**

	*x*	*y*	*z*	*U*_iso_*/*U*_eq_	
Pt1	0.098683 (11)	0.487050 (15)	0.187162 (8)	0.02001 (9)	
N1	0.1092 (2)	0.3711 (3)	0.23845 (16)	0.0200 (8)	
C1	0.1088 (3)	0.3805 (4)	0.29169 (19)	0.0230 (10)	
H1A	0.1042	0.4469	0.3070	0.028*	
N2	0.1029 (2)	0.3703 (3)	0.13593 (17)	0.0227 (9)	
C2	0.1149 (3)	0.2965 (4)	0.3246 (2)	0.0261 (11)	
H2A	0.1140	0.3054	0.3622	0.031*	
N3	0.1005 (2)	0.5974 (3)	0.24244 (18)	0.0247 (9)	
C3	0.1223 (3)	0.1981 (4)	0.30320 (17)	0.0188 (9)	
N4	0.0870 (2)	0.5961 (3)	0.13214 (18)	0.0261 (9)	
C4	0.1233 (3)	0.1908 (3)	0.24737 (17)	0.0203 (9)	
H4A	0.1280	0.1254	0.2310	0.024*	
C5	0.1174 (3)	0.2771 (4)	0.21635 (17)	0.0197 (9)	
C6	0.1182 (3)	0.2775 (4)	0.15764 (18)	0.0214 (10)	
C7	0.1341 (3)	0.1931 (3)	0.12624 (17)	0.0197 (9)	
H7A	0.1449	0.1288	0.1425	0.024*	
C8	0.1346 (3)	0.2010 (4)	0.07074 (18)	0.0235 (10)	
C9	0.1159 (3)	0.2968 (4)	0.0501 (2)	0.0282 (11)	
H9A	0.1142	0.3057	0.0125	0.034*	
C10	0.0999 (3)	0.3787 (4)	0.08242 (19)	0.0281 (12)	
H10A	0.0864	0.4429	0.0669	0.034*	
C11	0.1265 (3)	0.1023 (4)	0.33750 (18)	0.0224 (10)	
C12	0.2078 (3)	0.0461 (4)	0.32623 (19)	0.0261 (10)	
H12A	0.2115	0.0299	0.2882	0.039*	
H12B	0.2099	−0.0174	0.3470	0.039*	
H12C	0.2538	0.0903	0.3364	0.039*	
C13	0.0557 (3)	0.0306 (4)	0.3229 (2)	0.0274 (11)	
H13A	0.0624	0.0069	0.2861	0.041*	
H13B	0.0038	0.0675	0.3263	0.041*	
H13C	0.0557	−0.0285	0.3470	0.041*	
C14	0.1207 (3)	0.1287 (4)	0.39695 (19)	0.0315 (12)	
H14A	0.0716	0.1698	0.4034	0.047*	
H14B	0.1693	0.1677	0.4076	0.047*	
H14C	0.1177	0.0653	0.4178	0.047*	
C15	0.1569 (3)	0.1088 (4)	0.03663 (18)	0.0249 (10)	
C16	0.0911 (4)	0.0262 (4)	0.0438 (2)	0.0344 (13)	
H16A	0.0875	0.0072	0.0815	0.052*	
H16B	0.1057	−0.0344	0.0227	0.052*	
H16C	0.0382	0.0528	0.0317	0.052*	
C17	0.2397 (3)	0.0657 (5)	0.0539 (2)	0.0389 (13)	
H17A	0.2383	0.0504	0.0921	0.058*	
H17B	0.2826	0.1163	0.0468	0.058*	
H17C	0.2512	0.0027	0.0340	0.058*	
C18	0.1638 (4)	0.1369 (4)	−0.02260 (19)	0.0379 (14)	
H18A	0.1101	0.1584	−0.0359	0.057*	
H18B	0.1826	0.0771	−0.0428	0.057*	
H18C	0.2029	0.1932	−0.0269	0.057*	
C19	0.1018 (3)	0.6625 (4)	0.2714 (2)	0.0249 (11)	
C20	0.1019 (3)	0.7449 (5)	0.3107 (2)	0.0320 (13)	
H20A	0.1448	0.7945	0.3019	0.048*	
H20B	0.1124	0.7162	0.3461	0.048*	
H20C	0.0486	0.7793	0.3106	0.048*	
C21	0.0799 (3)	0.6560 (4)	0.1006 (2)	0.0282 (11)	
C22	0.0702 (4)	0.7321 (4)	0.0583 (2)	0.0435 (15)	
H22B	0.0863	0.7018	0.0241	0.065*	
H22C	0.1049	0.7916	0.0659	0.065*	
H22A	0.0129	0.7538	0.0565	0.065*	
F5	0.3387 (3)	0.4742 (3)	0.18538 (12)	0.0439 (9)	
F1	0.0964 (2)	0.5412 (3)	0.37735 (13)	0.0444 (9)	
F6	0.4162 (2)	0.3323 (3)	0.18232 (14)	0.0466 (9)	
F7	0.2907 (2)	0.3270 (3)	0.22056 (14)	0.0499 (9)	
F8	0.3013 (2)	0.3445 (3)	0.13080 (14)	0.0481 (9)	
F2	0.0499 (2)	0.5587 (3)	0.46122 (13)	0.0498 (9)	
F3	0.1841 (2)	0.5745 (3)	0.44502 (13)	0.0519 (10)	
F4	0.1237 (3)	0.4179 (3)	0.43888 (16)	0.0742 (14)	
B1	0.1145 (5)	0.5186 (6)	0.4330 (2)	0.0345 (16)	
B2	0.3361 (4)	0.3695 (5)	0.1792 (2)	0.0293 (13)	

### Atomic displacement parameters (Å^2^)

**Table:**

	*U*^11^	*U*^22^	*U*^33^	*U*^12^	*U*^13^	*U*^23^
Pt1	0.02479 (13)	0.01503 (13)	0.02021 (13)	−0.00068 (6)	−0.00068 (7)	0.00112 (6)
N1	0.025 (2)	0.018 (2)	0.018 (2)	−0.0017 (15)	−0.0023 (15)	−0.0012 (15)
C1	0.034 (3)	0.018 (2)	0.017 (2)	−0.0026 (19)	−0.0004 (19)	−0.0040 (19)
N2	0.031 (2)	0.016 (2)	0.022 (2)	−0.0061 (15)	−0.0026 (16)	0.0006 (16)
C2	0.037 (3)	0.023 (3)	0.019 (2)	−0.001 (2)	−0.003 (2)	−0.002 (2)
N3	0.026 (2)	0.021 (2)	0.028 (2)	0.0024 (15)	−0.0013 (16)	0.0027 (18)
C3	0.021 (2)	0.018 (2)	0.018 (2)	−0.0039 (18)	0.0009 (18)	0.0024 (17)
N4	0.030 (2)	0.018 (2)	0.031 (2)	0.0031 (16)	−0.0006 (17)	−0.0011 (18)
C4	0.023 (2)	0.022 (2)	0.016 (2)	−0.0032 (18)	−0.0004 (18)	−0.0033 (18)
C5	0.022 (2)	0.019 (2)	0.018 (2)	−0.0040 (17)	0.0009 (18)	−0.0023 (17)
C6	0.022 (2)	0.022 (2)	0.020 (2)	−0.0055 (18)	−0.0032 (18)	0.0021 (18)
C7	0.023 (2)	0.017 (2)	0.019 (2)	−0.0040 (18)	−0.0039 (18)	0.0020 (17)
C8	0.030 (3)	0.020 (2)	0.021 (2)	−0.0075 (19)	−0.002 (2)	0.0024 (18)
C9	0.044 (3)	0.027 (3)	0.013 (2)	−0.003 (2)	−0.003 (2)	0.0052 (19)
C10	0.043 (3)	0.023 (3)	0.018 (3)	−0.005 (2)	−0.008 (2)	0.0033 (19)
C11	0.029 (2)	0.022 (2)	0.017 (2)	0.002 (2)	−0.0029 (19)	0.0013 (18)
C12	0.029 (3)	0.024 (2)	0.026 (2)	0.007 (2)	0.003 (2)	0.009 (2)
C13	0.031 (3)	0.022 (2)	0.029 (3)	−0.006 (2)	0.001 (2)	0.007 (2)
C14	0.046 (3)	0.027 (3)	0.021 (3)	0.000 (2)	0.000 (2)	0.009 (2)
C15	0.034 (3)	0.025 (2)	0.016 (2)	−0.003 (2)	−0.0014 (19)	−0.0013 (19)
C16	0.049 (4)	0.024 (3)	0.030 (3)	0.001 (2)	−0.003 (2)	−0.007 (2)
C17	0.037 (3)	0.051 (4)	0.028 (3)	0.006 (3)	0.006 (2)	0.006 (3)
C18	0.066 (4)	0.035 (3)	0.013 (2)	0.006 (3)	−0.002 (2)	−0.003 (2)
C19	0.029 (3)	0.023 (3)	0.023 (3)	0.0030 (19)	0.002 (2)	−0.002 (2)
C20	0.041 (3)	0.022 (3)	0.032 (3)	0.001 (2)	0.000 (2)	−0.006 (2)
C21	0.032 (3)	0.023 (3)	0.029 (3)	0.008 (2)	0.005 (2)	0.005 (2)
C22	0.057 (4)	0.032 (3)	0.041 (4)	0.009 (3)	−0.006 (3)	0.022 (3)
F5	0.056 (2)	0.033 (2)	0.042 (2)	0.0095 (17)	0.0005 (15)	0.0018 (15)
F1	0.054 (2)	0.052 (2)	0.0273 (18)	0.0104 (16)	−0.0050 (14)	−0.0065 (17)
F6	0.0295 (17)	0.041 (2)	0.070 (3)	0.0038 (15)	0.0019 (15)	−0.0015 (17)
F7	0.0379 (19)	0.060 (2)	0.052 (2)	−0.0054 (16)	0.0043 (16)	0.0274 (19)
F8	0.049 (2)	0.049 (2)	0.046 (2)	−0.0100 (17)	−0.0084 (16)	−0.0111 (16)
F2	0.051 (2)	0.064 (3)	0.0348 (19)	−0.0122 (19)	0.0087 (16)	−0.0163 (17)
F3	0.046 (2)	0.074 (3)	0.0358 (19)	−0.0100 (19)	0.0048 (15)	−0.0153 (18)
F4	0.132 (4)	0.035 (2)	0.056 (3)	0.012 (2)	−0.024 (3)	−0.004 (2)
B1	0.035 (3)	0.046 (4)	0.023 (3)	0.014 (3)	−0.009 (3)	−0.026 (3)
B2	0.023 (3)	0.022 (3)	0.043 (4)	−0.005 (2)	0.003 (2)	0.005 (2)

### Geometric parameters (Å, º)

**Table:**

Pt1—N4	1.992 (4)	C13—H13B	0.9800
Pt1—N1	1.994 (4)	C13—H13C	0.9800
Pt1—N2	1.995 (4)	C14—H14A	0.9800
Pt1—N3	2.000 (5)	C14—H14B	0.9800
N1—C1	1.343 (6)	C14—H14C	0.9800
N1—C5	1.353 (6)	C15—C17	1.528 (7)
C1—C2	1.377 (7)	C15—C16	1.533 (7)
C1—H1A	0.9500	C15—C18	1.536 (6)
N2—C10	1.349 (6)	C16—H16A	0.9800
N2—C6	1.350 (6)	C16—H16B	0.9800
C2—C3	1.396 (7)	C16—H16C	0.9800
C2—H2A	0.9500	C17—H17A	0.9800
N3—C19	1.118 (7)	C17—H17B	0.9800
C3—C4	1.405 (6)	C17—H17C	0.9800
C3—C11	1.520 (6)	C18—H18A	0.9800
N4—C21	1.119 (7)	C18—H18B	0.9800
C4—C5	1.372 (6)	C18—H18C	0.9800
C4—H4A	0.9500	C19—C20	1.460 (7)
C5—C6	1.474 (6)	C20—H20A	0.9800
C6—C7	1.379 (6)	C20—H20B	0.9800
C7—C8	1.398 (6)	C20—H20C	0.9800
C7—H7A	0.9500	C21—C22	1.462 (7)
C8—C9	1.386 (7)	C22—H22B	0.9800
C8—C15	1.521 (7)	C22—H22C	0.9800
C9—C10	1.368 (8)	C22—H22A	0.9800
C9—H9A	0.9500	F5—B2	1.375 (7)
C10—H10A	0.9500	F1—B1	1.459 (7)
C11—C13	1.532 (7)	F6—B2	1.398 (7)
C11—C14	1.535 (6)	F7—B2	1.392 (6)
C11—C12	1.544 (7)	F8—B2	1.381 (7)
C12—H12A	0.9800	F2—B1	1.374 (7)
C12—H12B	0.9800	F3—B1	1.385 (8)
C12—H12C	0.9800	F4—B1	1.330 (8)
C13—H13A	0.9800		
			
N4—Pt1—N1	176.23 (16)	C11—C13—H13C	109.5
N4—Pt1—N2	95.81 (18)	H13A—C13—H13C	109.5
N1—Pt1—N2	80.47 (18)	H13B—C13—H13C	109.5
N4—Pt1—N3	88.22 (19)	C11—C14—H14A	109.5
N1—Pt1—N3	95.53 (18)	C11—C14—H14B	109.5
N2—Pt1—N3	175.27 (16)	H14A—C14—H14B	109.5
C1—N1—C5	119.5 (4)	C11—C14—H14C	109.5
C1—N1—Pt1	125.0 (3)	H14A—C14—H14C	109.5
C5—N1—Pt1	115.6 (3)	H14B—C14—H14C	109.5
N1—C1—C2	121.7 (5)	C8—C15—C17	110.1 (4)
N1—C1—H1A	119.2	C8—C15—C16	108.8 (4)
C2—C1—H1A	119.2	C17—C15—C16	109.2 (4)
C10—N2—C6	118.8 (4)	C8—C15—C18	112.0 (4)
C10—N2—Pt1	125.4 (4)	C17—C15—C18	107.4 (4)
C6—N2—Pt1	115.5 (3)	C16—C15—C18	109.4 (4)
C1—C2—C3	120.4 (5)	C15—C16—H16A	109.5
C1—C2—H2A	119.8	C15—C16—H16B	109.5
C3—C2—H2A	119.8	H16A—C16—H16B	109.5
C19—N3—Pt1	176.6 (4)	C15—C16—H16C	109.5
C2—C3—C4	116.7 (4)	H16A—C16—H16C	109.5
C2—C3—C11	122.8 (4)	H16B—C16—H16C	109.5
C4—C3—C11	120.6 (4)	C15—C17—H17A	109.5
C21—N4—Pt1	178.7 (5)	C15—C17—H17B	109.5
C5—C4—C3	120.6 (4)	H17A—C17—H17B	109.5
C5—C4—H4A	119.7	C15—C17—H17C	109.5
C3—C4—H4A	119.7	H17A—C17—H17C	109.5
N1—C5—C4	121.2 (4)	H17B—C17—H17C	109.5
N1—C5—C6	114.0 (4)	C15—C18—H18A	109.5
C4—C5—C6	124.8 (4)	C15—C18—H18B	109.5
N2—C6—C7	121.3 (4)	H18A—C18—H18B	109.5
N2—C6—C5	114.0 (4)	C15—C18—H18C	109.5
C7—C6—C5	124.7 (4)	H18A—C18—H18C	109.5
C6—C7—C8	120.8 (4)	H18B—C18—H18C	109.5
C6—C7—H7A	119.6	N3—C19—C20	177.7 (6)
C8—C7—H7A	119.6	C19—C20—H20A	109.5
C9—C8—C7	116.0 (4)	C19—C20—H20B	109.5
C9—C8—C15	123.7 (4)	H20A—C20—H20B	109.5
C7—C8—C15	120.3 (4)	C19—C20—H20C	109.5
C10—C9—C8	121.6 (5)	H20A—C20—H20C	109.5
C10—C9—H9A	119.2	H20B—C20—H20C	109.5
C8—C9—H9A	119.2	N4—C21—C22	178.4 (6)
N2—C10—C9	121.4 (5)	C21—C22—H22B	109.5
N2—C10—H10A	119.3	C21—C22—H22C	109.5
C9—C10—H10A	119.3	H22B—C22—H22C	109.5
C3—C11—C13	109.4 (4)	C21—C22—H22A	109.5
C3—C11—C14	111.3 (4)	H22B—C22—H22A	109.5
C13—C11—C14	108.9 (4)	H22C—C22—H22A	109.5
C3—C11—C12	109.0 (4)	F4—B1—F2	113.9 (7)
C13—C11—C12	108.5 (4)	F4—B1—F3	113.8 (5)
C14—C11—C12	109.7 (4)	F2—B1—F3	108.6 (4)
C11—C12—H12A	109.5	F4—B1—F1	109.1 (5)
C11—C12—H12B	109.5	F2—B1—F1	105.1 (5)
H12A—C12—H12B	109.5	F3—B1—F1	105.6 (6)
C11—C12—H12C	109.5	F5—B2—F8	110.2 (5)
H12A—C12—H12C	109.5	F5—B2—F7	109.1 (5)
H12B—C12—H12C	109.5	F8—B2—F7	110.0 (4)
C11—C13—H13A	109.5	F5—B2—F6	108.0 (5)
C11—C13—H13B	109.5	F8—B2—F6	110.7 (5)
H13A—C13—H13B	109.5	F7—B2—F6	108.7 (5)
